# Relative Telomere Length in Blood Leukocytes of Patients with Anterior Cruciate Ligament Injury: A Pilot Study

**DOI:** 10.5704/MOJ.1903.001

**Published:** 2019-03

**Authors:** P Daechavijit, J Siridonthanakasem, P Wongsupha, P Yuktanandana, S Honsawek

**Affiliations:** Osteoarthritis and Musculoskeletal Research Unit, Chulalongkorn University, Bangkok, Thailand; * Department of Orthopaedics, Chulalongkorn University, Bangkok, Thailand

**Keywords:** anterior cruciate ligament injury, contact sport, non-contact sport, relative telomere length

## Abstract

**Introduction:** Anterior cruciate ligament (ACL) tear is the most common knee ligament injury, especially in athletes. The objective of this study was to investigate relative telomere length (RTL) in blood leukocytes of patients with ACL injury compared with that of controls.

**Materials and Methods:** A total of 187 subjects were invited to participate in this study. Ninety-two patients with clinically diagnosed ACL rupture were enrolled. Ninety-five age and gender-matched healthy controls were also recruited. Blood leukocyte RTL were analysed using quantitative real-time polymerase chain reaction.

**Results:** Patients with ACL rupture had significantly longer relative telomere length than healthy controls (*P*=0.002). The patients with ACL rupture were classified into two groups according to the sport history of patients which are contact sports and non-contact sports. RTL in patients with non-contact sports was significantly greater than those with contact sports (*P*=0.006). Moreover, RTL was inversely correlated with body mass index of patients with ACL injury (*r*=-0.34, *P*=0.001). Logistic regression analysis indicated that long RTL was associated with a higher risk of ACL rupture.

**Conclusion:** The present study showed that subjects with ACL rupture had significantly greater telomere length compared with their age and gender-matched controls. This finding may result from the increases in physical activity and overexpression of telomerase which acts as a protective mechanism against ACL injury. RTL in blood leukocytes is associated with a risk of ACL rupture.

## Introduction

Anterior cruciate ligament (ACL) is located diagonally across the knee joint, attaching tibia and femur. The principal mechanical function of the ACL is to provide the rotational stability of a knee and to prevent excessive anterior tibial translation in various degrees of flexion. The anterior cruciate ligament is one of the most commonly injured structures in the knee. Disruption of the ACL results in functional knee instability, meniscal injury, and articular cartilage damage, knee pain, and poor life quality^1^.

The pathogenesis of ACL injuries has pointed mainly on mechanism of trauma, patient gender, and anatomic variations as predisposing causes^2^. Most ACL tears occur as a result of a non-contact valgus hyperextension mechanism, even though direct trauma could also be causative^3^. Although traumatic injuries to the knee leading to ACL ruptures are related to secondary knee osteoarthritis (OA), a number of extrinsic and intrinsic factors predispose an individual toward an ACL tear. Intrinsic risk factors include age, gender, anatomic variations, neuromuscular deficits, biomechanical factors, hormonal status, and genetic factors^4^.

Telomeres, the terminal repeated DNA sequences of TTAGGG and related proteins, are located at the chromosome ends. Telomere length represents the biological age of cells because their telomeres become shorter each time cells divide. Telomeres protect chromosomes against genomic instability by shielding chromosome ends from deterioration, fusion, and atypical recombination.

In recent years, reports revealed that there was no relationship between the age of the subjects and mean telomere length in ACL. Additionally, they showed no differences in relative telomere length (RTL) between the injured proximal part and the non-injured distal portion of anterior cruciate ligament^5^. Therefore, the genetic polymorphism may play a role in the mechanism related with telomere length in patients with ACL injury.

Epidemiologic studies have revealed that the abnormal telomere length in leukocytes is associated with some physical abilities and age-related disorders^6^. However, the association between leukocyte telomere length and ACL injury has not been examined. We postulated that relative telomere length in blood leukocytes of patients with ACL rupture would be lower than that of healthy controls.

The objective of this study was to investigate RTL in blood leukocytes of patients with ACL rupture compared with that of controls and determine the possible association between RTL in blood leukocytes and the ACL injury.

## Materials and Methods

This case-control study was approved by the Institutional Review Board on Human Research of our hospital. The present study was conducted in accordance with the guidelines of the Declaration of Helsinki. All subjects gave written informed consent prior to their participation in this study. A total of 187 subjects were recruited in the current study.

Ninety-two patients with clinically diagnosed ACL ruptures were enrolled from the Sport Medicine Clinics at our hospital. Additionally, 95 healthy volunteers without any self reported history of ACL injury were mainly recruited from medical students and colleagues in our hospital. The ACL subjects and healthy participants were age and gender matched. We classified patients with ACL rupture according to the mechanism of injury into contact sports and non-contact sports.

Genomic DNA was extracted directly from peripheral blood leukocytes using Illustra Blood GenomicPrep Mini Spin Kit according to the instruction of DNA extraction kit [GE Healthcare, Buckinghamshire, UK]. Leukocyte relative telomere length was assessed using a modified version of quantitative real-time polymerase chain reaction (qPCR) originally described by Cawthon^7^. The real-time PCR was conducted in a 96-well ABI StepOnePlus^TM^ Real-time PCR System [Applied Biosystems, Grand Island, USA]. To assess PCR efficiency, the standard curve of the reference sample was generated. The reference DNA sample was diluted to generate a 5-point standard curve, between 12.5 and 0.78 ng DNA by using a 2-fold serial dilution. The PCR reaction mixture (10 μL) for both telomere and *36B4* gene amplification consisted of 1X Power SYBR® Green PCR Master Mix [Applied Biosystems, Warrington, UK], 200 nmol/L each telomere or single copy gene (*36B4*) primers, and 3.12ng genomic DNA. Human gene-specific primer sequences were as follows:

Telomere forward primer: 5’-CGGTTT[GTTTGG]_5_GTT-3’

Telomere reverse primer: 5’-GGCTTG[CCTTAC]_5_CCT-3’

*36B4* forward primer: 5’-CAGCAAGTGGGAAGGTGTAATCC-3’

*36B4* reverse primer: 5’-CCCATTCTATCATCAACGGGTACAA-3’

The thermal cycling conditions for both telomere and *36B4* were enzyme activation at 95ºC for 10 minutes, followed by 40 cycles of denaturation at 95ºC for 15 seconds and annealing-extension at 54ºC for 1 minute. All samples were performed in duplicate for both telomere and *36B4* reactions in the same run. The reference and quality control samples were added in every run.

The CT values from qPCR were exported as Excel files, and relative telomere lengths (RTLs) were computed using the comparative method. Briefly, the ratio of telomeric repeats copy number to a single copy (*36B4* on chromosome 12) reference gene (T/S Ratio, ΔCt) was determined for each sample by subtracting the average threshold cycle (CT) value of *36B4* from the average CT value of telomere. The relative T/S ratio (ΔΔCT), which represents RTL values, was then calculated by subtracting the T/S ratio of a reference sample from the T/S ratio of each experimental sample, and then exponentiating (2^-ΔΔCT^).

Statistical analyses were performed using the Statistical Package for the Social Sciences (SPSS) software version 22.0 for Windows. Tests of normality and test of homogeneity of variances were used to analyse the subject’s age, body mass index (BMI), and relative telomere length in blood leukocytes. The differences in the distribution of baseline characteristics between ACL patients and controls were compared using unpaired t-test for continuous variables, and Chi-square test for categorical variables. The Kolmogorov-Smirnov test and quantile-quantile plot were used to assess whether relative telomere length was normally distributed. Given that relative telomere length data were not normally distributed, non-parametric continuous variables were expressed as median (interquartile range (IQR)) and compared using Mann-Whitney U test. Spearman’s rank correlation coefficient test was used to define the relationship between RTL and BMI. The associations of RTL with the risk of ACL rupture were measured by applying univariate and multivariate logistic regression analyses to determine the roles of confounding factors. A P-value<0.05 (based on a two-tailed test) was considered statistically significant.

## Results

**Fig. 1:**
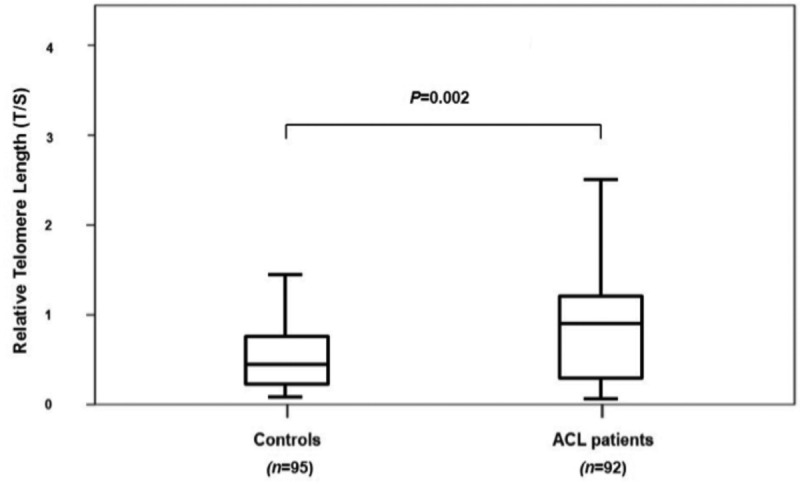
Box-plot illustrating relative telomere length distribution between ACL rupture subjects and healthy controls. The line through the middle of the boxes represents the median of T/S value and the top and bottom of each box represents the first and third quartiles.

A total of 187 participants were prospectively registered in the case-control study. There were no significant differences in age, weight, height, and body mass index (BMI) between ACL injury patients and healthy controls (Table I). The median RTL in blood leukocytes of patients with ACL injury was 0.941 (inter quartile range, IQR: 0.317, 1.150), and the median RTL in blood leukocytes of age-matched healthy controls was 0.422 (0.252, 0.711) (Fig. 1). The relative telomere length in blood leukocytes of patients with ACL injury was significantly higher than that in healthy controls (*P*=0.002) (Fig. 1).

**Table I:** Baseline characteristics of ACL injury patients and healthy controls

Characteristics	ACL rupture	Controls	p-Value
n	92	95	-
Age (years)	25.9±5.2	28.4±8.6	0.5
Weight (kg)	63.5±10.8	65.8±9.5	0.4
Height (cm)	168.9±7.6	169.5±6.6	0.5
Body mass index (kg/m^2^)	22.7±4.5	22.9±3.2	0.6

**Fig. 2:**
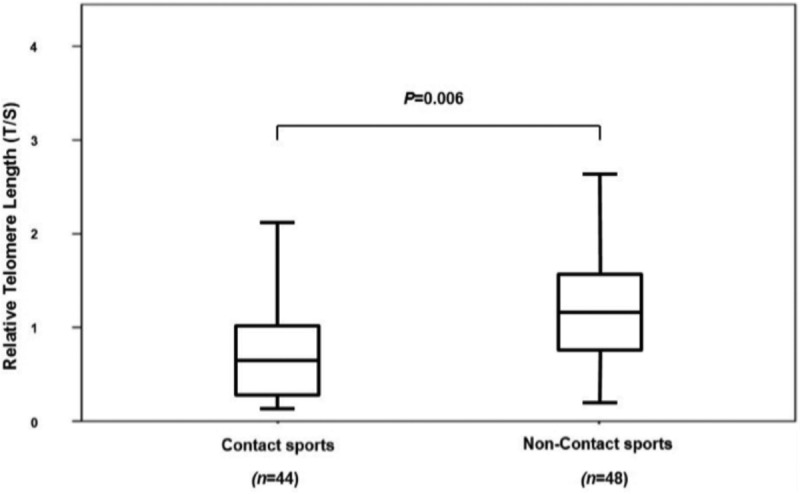
Box-plot illustrating relative telomere length distribution in ACL rupture subjects between contact sports and non-contact sport. The line through the middle of the boxes represents the median of T/S value and the top and bottom of each box represents the first and third quartiles.

Subsequently, the ACL injury patients were classified into two groups according to the mechanism of ACL injury: contact sports and non-contact sports. The median RTL in blood leukocytes of patients with non-contact sports was 1.172 (0.787, 1.495), and the median RTL in blood leukocytes of patients with contact sports was 0.592 (0.258, 1.012) (Fig. 2). The patients with non-contact sports seemed to have significantly longer RTL than those with contact sports (*P*=0.006) (Fig. 2). Further analysis showed that RTL in blood leukocytes of ACL injury patients was inversely correlated with their BMI (*r*=-0.34, *P*=0.001).

Since telomere length can be influenced by age, gender, and BMI, we performed logistic regression analysis to control the role of confounding variables. After adjusting for age, gender and BMI, RTL in subjects with ACL rupture was significantly longer than that of the controls by an average of 2.109 units (95% CI: 0.833 to 5.342, *P*=0.01). The RTL of participants were separated into short RTL and long RTL groups, based on the median distribution of RTL in healthy controls. The subjects with long RTL had a significantly increased risk of ACL rupture, as compared to subjects with short RTL in both univariate (unadjusted OR: 1.826, 95% CI: 1.016 to 3.280, *P*=0.02) and multivariate analysis (adjusted OR: 2.301, 95% CI: 0.840 to 6.301, *P*<0.05) (Table II). We further classified study subjects into three groups according to the tertile of RTL values in controls and investigated a significant dose-response association between long RTL and higher risk of ACL rupture. Specifically, using the first tertile (shortest) as the reference group, the odds ratios (OR) for the second and third tertiles were 0.872 (95% CI: 0.389 to 1.952, *P*=0.37) and 2.519 (95% CI: 1.233 to 5.144, *P*=0.005), respectively, in unadjusted univariate analysis and 1.298 (95% CI: 0.301 to 5.595, *P*=0.36) and 3.797 (95% CI: 1.054 to 13.682, *P*=0.02), respectively, in multivariate analysis. The *P* trend was less than 0.05 in both analyses, suggesting evidence for a dose-response effect of long RTL-related higher risk of ACL rupture.

**Table II:** Logistic regression analysis of association between RTL and risk of ACL rupture

RTL	ACL rupture	Controls	Unadjusted OR (95% CI)	p-Value	Adjusted* OR (95% CI)	p-Value
Overall	92	95	2.831 (1.563-5.061)	0.001	2.109 (0.833-5.342)	0.01
By median						
Short	33	48	1 (reference)		1 (reference)	
Long	59	47	1.826 (1.016-3.280)	0.02	2.301 (0.840-6.301)	<0.05
By tertile						
1st tertile	20	31	1 (reference)		1 (reference)	
2nd tertile	18	32	0.872 (0.389-1.952)	0.37	1.298 (0.301-5.595)	0.36
3rd tertile	54	32	2.519 (1.233-5.144)	0.005	3.797 (1.054-13.682)	0.02
P trend				<0.05		<0.05

ACL=anterior cruciate ligament; RTL=relative telomere length; OR=odds ratio; CI=confidence interval *Adjusted by age, gender and body mass index

## Discussion

Anterior cruciate ligament (ACL) is one of the most commonly injured ligaments in the knee, especially in athletes. ACL ruptures are considered to be the most severe joint injury in sports. However, the exact etiologies of ACL injuries are not fully understood. In recent years, a growing interest in telomere length has led to a number of studies investigating relative telomere length in various disease conditions including osteoarthritis, lumbar spinal stenosis, and rheumatological disorders^8-10^. However, to date, whether blood leukocyte RTL associate with the risk of ACL rupture has never been investigated.

To the best of our knowledge, this is the first study to assess the relative leukocyte telomere length in patients with ACL rupture. Our study reveals a significant difference in the mean relative telomere length of peripheral blood leukocytes in ACL rupture patients and controls. ACL rupture patients had significantly longer telomere than healthy controls. The longer telomere length observed in the ACL injury subjects in our study may be a result of increased telomerase expression and activity in leukocytes. This may result from overexpression of telomerase which acts as a protective mechanism against ACL injury. The adding back telomeric repeats is the main function of telomerase and therefore would lead to longer telomere length in subjects with ACL rupture. Furthermore, our data suggest that RTL of patients with ACL injury was negatively correlated with BMI. This result suggests the more patients overweight, the shorter RTL they would have.

Recently, Ponsot and colleagues has conducted the telomere length in ACL samples of patients with ACL rupture^5^. They indicated that mean telomere length values between the three ACL regions (proximal, middle, and distal ACL tissues) were not significantly different, whereas our findings found that blood leukocyte telomere length was significantly longer in patients with ACL rupture than healthy controls. This could be explained by the difference in type of samples, method analyses, and sample size. Previous study investigated telomere length in ACL tissue samples using telomere restriction fragment assay^5^, while this study measured relative telomere length in blood leukocytes using quantitative real-time PCR analysis.

The underlying molecular mechanisms behind the connection of long RTL in leukocytes and ACL rupture remain incompletely understood. Telomere length of proliferative tissues, such as leukocytes, is longest at birth and shortening depends upon genetic and lifestyle factors. Accumulating evidence has unveiled a positive influence of physical activity levels on leukocyte telomere length^11,12^. An increase in moderate-intensity physical activity correlates with longer leukocyte telomere length^13^. ACL injury almost exclusively occurs in athletes who engaged in less sitting and were more physically active compared with their non-athletic participants. Although our findings suggest that ACL injury patients were associated with longer RTL, the molecular mechanisms leading to longer leukocyte telomeres in these subjects are unclear. Upregulation of telomerase could be a likely mechanism of longer RTL in our participants.

In this study, we observed that the patients with contact sports had significantly shorter RTL than those with non-contact sports. Telomere length may be influenced by various factors including oxidative stress and inflammation. The explanation for short RTL in subjects with contact sport might be attributed to oxidative stress and inflammation. Oxidative stress has been demonstrated to shorten telomere length in cells cultured *in vitro*^14^. The decrease of telomere length may be accelerated by telomeric DNA damage due to oxidative stress, chronic inflammation, increased cellular turnover, or defects in telomere repair^15^. Oxidative stress known to affect telomere biology was not measured and could have affected the findings in this study. Further studies will focus on combining data with oxidative stress markers in relation to telomere length in blood leukocytes and tissue samples.

There are some limitations in this study. Since leukocyte telomere length was measured but telomerase was not, the effect of telomerase relating to ACL rupture in this study remains undetermined. In addition, all participants were Thai, and it is not known whether these findings are generalisable among other ethnic groups. Moreover, the present study was cross-sectional in nature and thus we were unable to assign direct causative nature to the correlation between telomere length and the severity of ACL injury. Accordingly, the mechanism behind the relation of longer leukocyte telomere length remains a mystery and requires further investigation. Some sources of bias have been identified in the current study, and the findings cannot be extrapolated to the general population. More studies are needed in larger populations of different ethnicities. More studies in gene-gene interactions, gene expression, and specific protein that triggers changes in the RTL may delineate the exact role of telomere and gene expression in ACL tears.

## Conclusion

The present study demonstrates longer relative leukocyte telomere length in patients with ACL rupture compared with healthy controls. Additionally, patients with non-contact sports had significantly greater telomere length than those with contact sports. These findings suggest that long telomere length may be due to the increases in telomerase expression for telomere maintenance in leukocytes of athletes with ACL rupture. The consequences of oxidative stress and cellular senescence on the course and outcomes of ACL injury remain to be elucidated in larger prospective studies.
